# Smoking predicts brain atrophy in 10,134 healthy individuals and is potentially influenced by body mass index

**DOI:** 10.1038/s44400-025-00024-0

**Published:** 2025-07-23

**Authors:** Somayeh Meysami, Saurabh Garg, Sam Hashemi, Nasrin Akbari, Ahmed Gouda, Yosef Gavriel Chodakiewitz, Thanh Duc Nguyen, Rajpaul Attariwala, Kellyann Niotis, David A. Merrill, Cyrus A. Raji

**Affiliations:** 1 Pacific Brain Health Center, Pacific Neuroscience Institute and Foundation, Santa Monica, CA USA; 2 Saint John’s Cancer Institute at Providence Saint John’s Health Center, Santa Monica, CA USA; 3 Prenuvo, Vancouver, BC Canada; 4 Prenuvo, Los Angeles, CA USA; 5 Voxelwise Imaging Technology, Vancouver, BC Canada; 6 AIM Medical Imaging, Vancouver, BC Canada; 7 Early Medical, Austin, TX USA; 8 The Institute for Neurodegenerative Diseases-Florida, Boca Raton, FL USA; 9 Preventive Neurology, New York, NY USA; 10 Department of Psychiatry and Biobehavioral Sciences, David Geffen School of Medicine at University of California Los Angeles, Los Angeles, CA USA; 11 Washington University in St Louis Mallinckrodt Institute, St. Louis, MO USA

**Keywords:** Diagnostic markers, Alzheimer's disease

## Abstract

Cigarette smoking is a risk factor for Alzheimer’s and vascular dementia, but its impact on brain volume loss, a neurodegeneration biomarker on MRI, is unclear. In total, 10,134 participants from 4 sites were scanned with a whole-body 1.5 T MRI protocol with separate dedicated structural neuroimaging with 3D T1 MPRAGE sequences. Smokers versus non-smokers were compared by gray and white matter volumes normalized to total intracranial volume using a two-tailed *t*-test. Smokers had lower normalized gray (*t* = −7.806e+00, *p* = 6.508e-15) and white matter volumes (*t* = −7.374e + 00, *p* = 1.791e-13) compared to non-smokers. Adjusting for age, sex, study site, BMI, and multiple comparisons, higher pack years of smoking predicted volume loss in such regions as total gray matter volume, total white matter volume, temporal lobe, parietal lobe, hippocampus, precuneus, and posterior cingulate. The inclusion and exclusion of BMI from the model suggested an influence of this variable.

## Introduction

Alzheimer’s dementia (AD) is a progressive neurodegenerative disorder that affects cognitive function, memory, and behavior. The prevalence of dementia is increasing worldwide, and it is estimated that ~47 million people are affected by dementia globally, with 10 million new cases each year^1^. The initial understanding that close to half of dementia cases are preventable^2,3^ has since evolved to a detailed understanding of early, midlife, and late-life risk factors for dementia^4^. One late-life risk factor in late life is smoking, which is estimated to contribute up to 14% of dementia cases worldwide^4,5^. Smoking is known to increase the risk of cerebrovascular disease, including ischemic and hemorrhagic stroke, which are independent risk factors for dementia^6^. In addition, toxins in cigarette smoke can drive neuroinflammation^7^, a mechanism that has also been linked to AD^8^.

To understand how smoking can modify dementia risk, it is important to evaluate its influence on brain atrophy, and loss of brain tissue from shrinkage or death of neurons with reduced neuronal connections^9^. Brain atrophy is tracked on neuroimaging by volume loss on T1-weighted structural imaging and is distinct between aging, AD^10^, and other neurodegenerative as well as non-neurodegenerative disorders^11–13^. Additionally, cerebral volume loss on MRI has been characterized as an acceptable biomarker of neurodegeneration^14^. Thus, investigating the relationship between smoking and atrophy reflected by MRI-measured brain volume loss is important to determine how it modifies risk for cognitive decline and AD. However, such information is lacking in large populations.

The purpose of this work was to therefore test the hypothesis that both the history of smoking and pack years of smoking are related to a higher burden of brain atrophy at whole brain and regional lobar levels. We also hypothesize that smoking is related to lower volumes in brain regions affected by AD pathology, specifically the hippocampus, and precuneus. The secondary goal of this study is to determine if this influence is mediated by body mass index (BMI), given the known relationship between this variable and smoking^15^ as well as our prior work on obesity and brain atrophy^16,17^. We sought to test these hypotheses in large cohorts that are more likely to show generalizable results.

## Results

Compared to non-smokers, smokers were more likely to be older in age, women, and Caucasian, and have a higher BMI and increased rates of hypertension and type 2 diabetes mellitus (Table 1). The smoking group had a mean pack years of 11.93 ± 14.69.

**Table 1:** Participant summary information

Variable (*n* = 10,134)	Smoking^a^ (*n* = 3292)	Non-smoking (*n* = 6842)	*T*-statistic (*p* value)
**Age Mean** **±** **SD (Age range)**	54.7 ± 13.1 (Range: 20–97)	52.2 ± 12.8 (Range: 18–97)	9.16 (6.61e-20)
**Biological sex (men/women)**	51.2% (1686)/ 48.8% (1606)	52.9% (3619)/ 47.1% (3223)	−2.83 (4.73e-03)
**Race and ethnicity** Caucasian/Sub-Saharan African/East Asian/West Asian/South Asian/Latin American/Indigenous/Mixed/Not Sure	63.2% (2079)/0.2% (8)/6.3%(209)/2.5%(81)/4.4%(146)/1.6% (52)/0.02%(8)/18.2%(599)/2.9%(97)	58.6% (4002)/0.4% (28)/10% (681)/2.7% (185)/8.7% (593)/ 1.9% (131)/0.1% (4)/14.6% (999)/2.7% (184)	4.44 (9.21e-06) (for Caucasians)
**Body mass index** **Mean** **±** **SD (BMI range)**	26.84 ± 5.3 (6.2–73)	25.85 ± 5 (7–67.9)	8.53 (1.67e-17)
**Hypertension**	10.3% (340)	8.1% (551)	3.85 (1.18e-04)
**Type 2 diabetes**	4.6% (152)	3% (206)	2.64 (8.27e-03)

^a^Smokers are defined as any self-reported smoking history (i.e., a non-zero pack-year value). Non-smokers are defined as those reporting zero pack years (never smokers).

Table 2 demonstrates the results of a two-sample *t*-test comparing brain volumes between smoking and non-smoking.

**Table 2:** Comparison of normalized brain volumes between smokers and non-smokers

Brain region	Smoking mean volume ± standard deviation	Non-smoking mean volume ± standard deviation	*t*-statistic (*p* value^a^)
Gray Matter (GM)	0.24808 ± 0.038	0.25424 ± 0.037	−7.806 (6.508e-15)
White Matter (GM)	0.23363 ± 0.036	0.23915 ± 0.035	−7.374 (1.791e-13)
Lateral Ventricle (Ventricle)	0.01148 ± 0.006	0.01088 ± 0.006	4.783 (1.750e-06)
Frontal Lobe	0.06875 ± 0.011	0.07083 ± 0.011	−8.779 (1.908e-18)
Temporal Lobe	0.05243 ± 0.008	0.05379 ± 0.008	−7.826 (5.525e-15)
Parietal Lobe	0.04853 ± 0.008	0.04963 ± 0.008	−6.730 (1.787e-11)
Occipital Lobe	0.02362 ± 0.004	0.02400 ± 0.004	−4.728 (2.299e-06)
Hippocampus	0.00404 ± 0.001	0.00413 ± 0.001	−6.735 (1.731e-11)
Cerebellum	0.06571 ± 0.011	0.06687 ± 0.010	−5.294 (1.220e-07)
Cingulate	0.00298 ± 0.001	0.00306 ± 0.000	−7.008 (2.576e-12)
Precuneus	0.00891 ± 0.001	0.00903 ± 0.001	−3.814 (1.375e-04)

^a^Corrected for multiple comparisons.

**Fig. 1:**
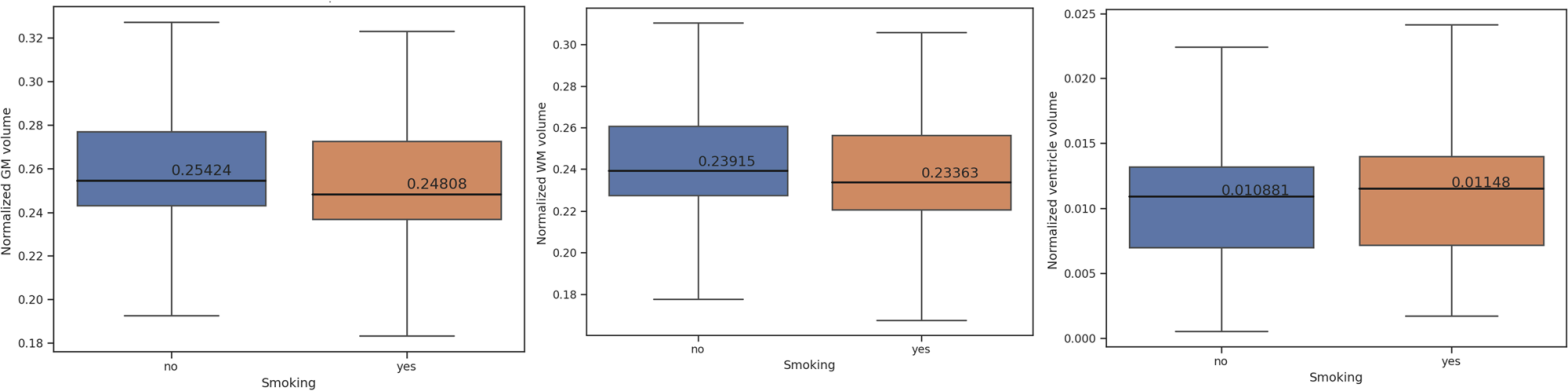
This figure shows comparisons of whole brain volumes between the smoking (orange boxplot) and non-smoking (blue boxplot) groups. Persons who smoke showed lower GM, WM, and ventricle volumes compared to non-smokers.

**Fig. 2:**
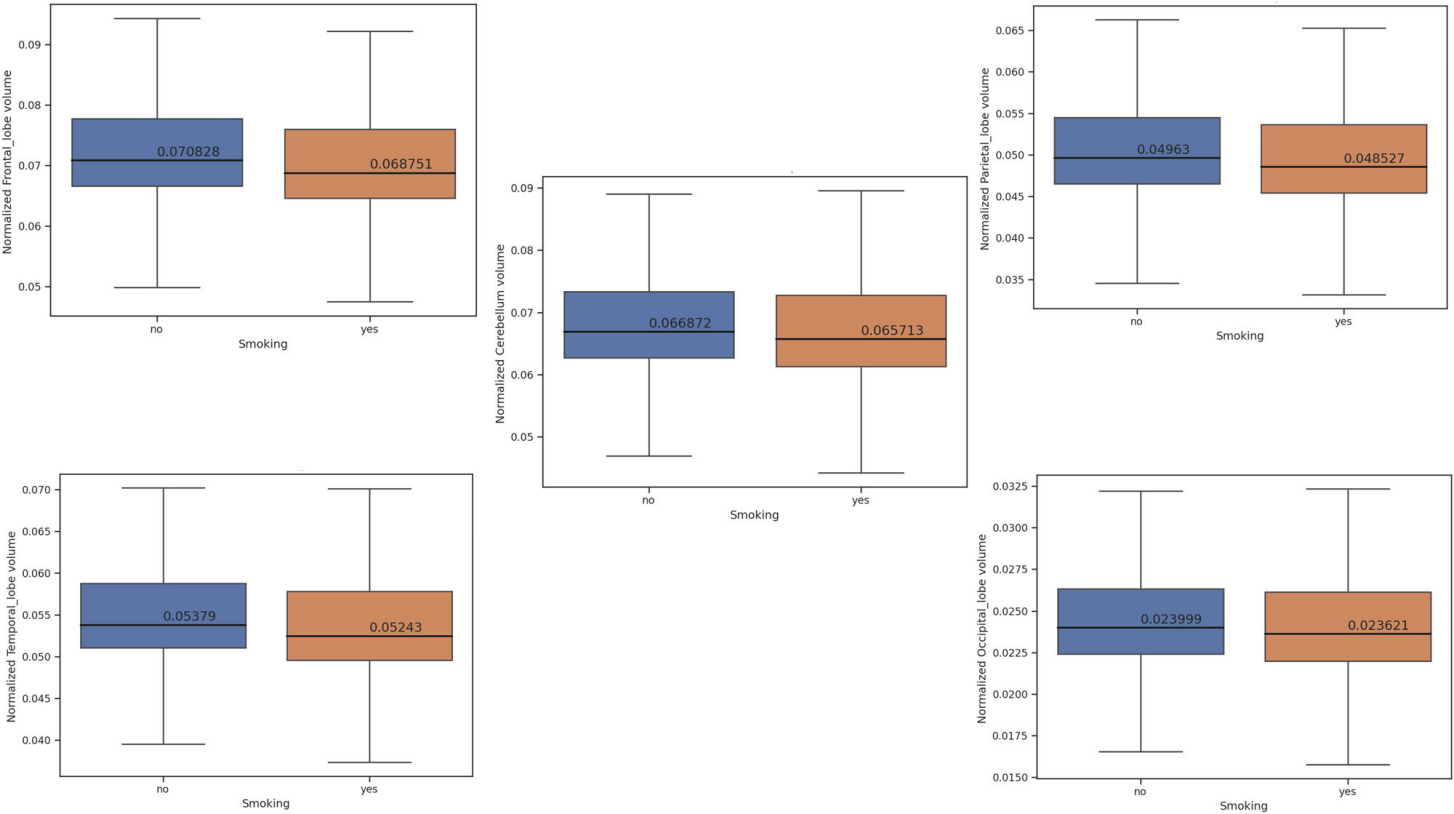
This figure demonstrates that lobar and cerebellar volumes were lower in the smoking compared to non-smoking groups.

**Fig. 3:**
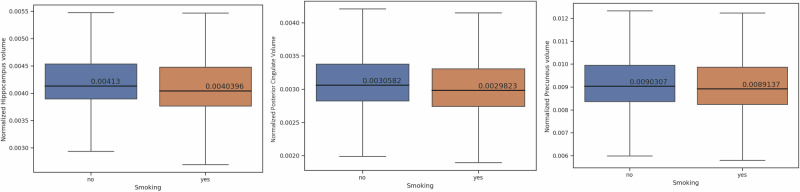
All of the AD risk regions compared were lower in the smoking group compared to the non-smoking group, as demonstrated in this figure.

Groupwise regional comparisons showing lower brain volumes in smoking versus non-smoking groups are shown in Figs. 1–3.

Table 3 shows the results from Model 1 (A) and Model 2 (B) of the regression results of pack years of smoking against quantified brain volumes.

**Table 3:** (A) Model 1 results on the regression of smoking pack years against brain volumes, adjusting for age, sex, and study site and (B) Model 2 results on the regression of smoking pack years against brain volumes, adjusting for age, sex, and BMI

Brain region	Standardized β coefficient (standard error)	*T*-statistic (*p* value^a^)	*R*^2^	Adjusted *R*^2^
**A**				
Gray Matter	−2.948e-04 (4.233e-05)	−6.963 (3.645e-12)	7.045e-03	6.900e-03
White Matter	−3.049e-04 (4.218e-05)	−7.228 (5.441e-13)	7.587e-03	7.442e-03
Lateral Ventricle	−1.053e-05 (6.179e-06)	−1.704 (8.844e-02)	4.247e-04	2.784e-04
Frontal Lobe	−9.153e-05 (1.233e-05)	−7.424 (1.275e-13)	8.002e-03	7.856e-03
Temporal Lobe	−6.680e-05 (9.292e-06)	−7.189 (7.201e-13)	7.508e-03	7.362e-03
Parietal Lobe	−5.128e-05 (8.733e-06)	−5.872 (4.498e-09)	5.021e-03	4.876e-03
Occipital Lobe	−2.026e-05 (4.464e-06)	−4.538 (5.782e-06)	3.004e-03	2.859e-03
Hippocampus	−4.722e-06 (7.358e-07)	−6.418 (1.476e-10)	5.991e-03	5.846e-03
Cerebellum	−6.750e-05 (1.213e-05)	−5.567 (2.695e-08)	4.515e-03	4.369e-03
Posterior Cingulate	−4.261e-06 (5.780e-07)	−7.372 (1.883e-13)	7.890e-03	7.745e-03
Precuneus	−6.618e-06 (1.687e-06)	−3.923 (8.845e-05)	2.247e-03	2.101e-03
**B**				
Gray Matter	−2.110e-04 (4.025e-05)	−5.242 (1.633e-07)	4.006e-03	3.860e-03
White Matter	−2.288e-04 (4.048e-05)	−5.653 (1.639e-08)	4.655e-03	4.510e-03
Lateral Ventricle	−7.494e-06 (6.160e-06)	−1.217 (2.238e-01)	2.166e-04	7.024e-05
Frontal Lobe	−6.797e-05 (1.177e-05)	−5.777e (7.959e-09)	4.860e-03	4.714e-03
Temporal Lobe	−4.851e-05 (8.840e-06)	−5.487 (4.226e-08)	4.387e-03	4.242e-03
Parietal Lobe	−3.577e-05 (8.387e-06)	−4.264 (2.033e-05)	2.654e-03	2.508e-03
Occipital Lobe	−1.240e-05 (4.289e-06)	−2.890e (3.863e-03)	1.221e-03	1.075e-03
Hippocampus	−3.441e-06 (7.080e-07)	−4.860 (1.201e-06)	3.445e-03	3.299e-03
Cerebellum	−4.602e-05 (1.165e-05)	−3.951 (7.869e-05)	2.279e-03	2.133e-03
Posterior Cingulate	−3.249e-06 (5.560e-07)	−5.842 (5.380e-09)	4.971e-03	4.825e-03
Precuneus	−4.091e-06 (1.640e-06)	−2.495 (1.262e-02)	9.101e-04	7.639e-04

^a^Corrected for multiple comparisons.

A Pearson bivariate correlation shows that higher BMI is related to increased smoking pack years (*r* = 0.16, *p* = 8.55e-19). In comparing Model 1 and Model 2, the inclusion of BMI in Model 2 demonstrates a systematic weakening of statistical significance and explanatory power in all eleven regions evaluated on structural brain MRI. These results which are shown in Table 4 demonstrate the respective *p* value results, the explanatory power, and the difference in the adjusted R^2^ in both models. The observed reduction in effect size, statistical significance, and variance explained (*R*^2^) upon inclusion of BMI suggests a potential mediating role of BMI in the relationship between the increase in smoking pack years and reduced brain volumes.

**Table 4:** Comparison of Model 1 and Model 2 results with the inclusion of BMI

Brain region	*p* value^a^ Model 1	*p* value^a^ Model 2	*R*^2^ Model 1	*R*^2^ Model 2	Δ*R*^2^
Gray Matter	3.645e-12	1.633e-07	7.045e-03	4.006e-03	−3.039e-03
White Matter	5.441e-13	1.639e-08	7.587e-03	4.655e-03	−2.932e-03
Lateral Ventricle	8.844e-02	2.238e-01	4.247e-04	2.166e-04	−2.081e-04
Frontal Lobe	1.275e-13	7.959e-09	8.002e-03	4.860e-03	−3.142e-03
Temporal Lobe	7.201e-13	4.226e-08	7.508e-03	4.387e-03	−3.121e-03
Parietal Lobe	4.498e-09	2.033e-05	5.021e-03	2.654e-03	−2.367e-03
Occipital Lobe	5.782e-06	3.863e-03	3.004e-03	1.221e-03	−1.783e-03
Hippocampus	1.476e-10	1.201e-06	5.991e-03	3.445e-03	−2.546e-03
Cerebellum	2.695e-08	7.869e-05	4.515e-03	2.279e-03	−2.236e-03
Posterior Cingulate	1.883e-13	5.380e-09	7.890e-03	4.971e-03	−2.919e-03
Precuneus	8.845e-05	1.262e-02	2.247e-03	9.101e-04	−1.337e-03

^a^Corrected for multiple comparisons.

## Discussion

In this work, we demonstrated that both a history of smoking and higher pack years of smoking relate to lower global, regional, and AD-relevant brain regions. Our data also preliminarily suggest a potential mediating role of BMI. Our work has implications for AD prevention as both obesity and smoking are respective midlife and late-life risk factors for dementia, including AD^4^. A meta-analysis confirmed that smoking increases the risk for AD and vascular dementia, with an increased risk modification profile in APOE4 positive individuals^18^.

The mechanisms by which smoking increases the risk for dementia remain incompletely understood, with oxidative stress and related generation of reactive oxygen species as one potential mechanism^19,20^. Nicotine from cigarette smoke has also been suggested as promoting amyloid-beta deposition through increased mRNA expression of the amyloid precursor protein^21^. Smoking has also been shown to induce amyloidosis, neuroinflammation, and tau hyperphosphorylation in a transgenic mouse model of AD^22^. Cigarette smoking can also impact the cerebral vascular system through several mechanisms. Smoking increases the risk of non-amyloid microangiopathy, which can lead to vascular cognitive impairment and vascular dementia. Additionally, nicotine and tobacco smoke have been shown to induce both vasoconstrictive and vasodilatory effects that can lead to chronic hypoperfusion^23^. Smoking-induced cerebral hypoperfusion occurs at the molecular level by impaired synthesis of nitric oxide through endothelial nitric oxide synthase^24^. A study using arterial spin-labeled MRI comparing 34 smokers with 27 non-smokers demonstrated hypoperfusion in posterior cortical regions, including those affected by AD pathology^25^. In a larger midlife cohort of 522 individuals from the CARDIA study, former smokers had lower cerebral blood flow in the precuneus, cuneus, occipital lobes, putamen, and insula^26^. The structural basis of such reduced cerebral perfusion in smokers was confirmed in a study of 15 male smokers less than 45 years of age who received magnetic resonance angiography, showing diminished distal cerebral vasculature in the middle cerebral artery territory^27^.

Reduced cerebral perfusion in turn can drive brain atrophy. Chronic brain hypoperfusion is linked to brain atrophy and increased risk for AD^28,29^. Also, multiple studies have shown an increased burden of brain atrophy in smokers. While the CARDIA study showed volume loss in total gray matter, lobar, cingulate, and amygdala volumes in current compared to never smokers, they did not show any volumetric differences between former and never smokers^30^, making it less likely we would have detected such differences in our study as we did not evaluate former smokers. One study used Freesurfer to analyze brain 4 T MRI scans comparing brain volumes in 41 non-smokers versus 41 smokers between 22 and 70 years of age^31^. The results showed lower brain volumes in relation to higher pack years, specifically in composite AD regions: the precuneus, lateral orbital frontal cortex, fusiform gyrus, middle temporal, and inferior parietal cortex. A 4-year longitudinal study of 1111 individuals aged 65–80 years found that smoking predicted increased hippocampal atrophy but not an increased rate of white matter volume loss^32^. However, these studies did not typically examine the influence of other covariates such as obesity as evaluated with BMI. Persons with higher BMI are more likely to smoke in part because this approach is thought to aid weight loss^33^. Concurrently, increased BMI has been linked to increased brain volume loss for example in the UK Biobank study^34^. In this context, the results from our statistical models show a reduced number of regions that are statistically significantly related to pack years when BMI is accounted for, suggesting a mediation effect^35^.

The advantage of this study is the availability of smoking history and pack years in a large cohort with the availability of quantitative structural brain imaging. Another advantage of this work includes the ability to measure regional brain volume at risk for Alzheimer’s disease pathology, such as the hippocampus, posterior cingulate, and precuneus. The availability of other covariates, such as BMI, allowed for related exploratory testing regarding this variable. The main drawback of this work is the cross-sectional design that precludes conclusions regarding causation. Although including BMI as a covariate in our models resulted in reduced statistical significance, effect sizes, and variance explained (*R*^2^) in the relationship between smoking pack years and brain volume—suggesting influence of BMI by way of potential mediation—we did not perform explicit mediation or moderation (i.e., interaction) analyses. This decision also reflects our study’s cross-sectional design, which lacks the temporal resolution required for reliable mediation or moderation testing. Thus, the influence of BMI by potential mediation is exploratory, warranting future confirmatory analyses in longitudinally designed studies with related structural equation modeling methods beyond the scope of this current work. Also, while we do show increased volume loss in AD-related brain regions even when accounting for age, sex, and BMI, we lack molecular AD biomarkers such as amyloid and tau to better understand these relationships. We also lacked cognitive evaluations to link the reduced brain volumes with smoking to any cognitive dysfunction. However, future work holds promise for bridging these gaps as fluid biomarkers become increasingly available^36^. Given the relationship between increased smoking pack years and larger white matter hyperintensities^37^ as well as white matter hyperintensities and brain atrophy^38^, future work should also investigate potential mediating effects of white matter hyperintensity volume and brain atrophy with respect to smoking history and pack years.

Understanding who will develop AD and dementia requires investigation of related modifiers and risk factors. The benefit of this knowledge is that it can help optimize brain health in such individuals that are becoming increasingly relevant with the development of new anti-AD therapies. Beyond this important clinical need, optimizing brain health also holds promise for maintaining cognition across the lifespan.

## Methods

### MR imaging participants

Institutional Review Board approval was granted for this study across all sites of the same institution and managed by an external agency (AdvarraWPBP-001, #Pro00046779) with waiver of informed consent and compliance with all regulations. MR imaging was performed on 1.5 T Philips Ingenia Ambition, Siemens Espree, and Aera scanners in the following sites: Vancouver, BC, Canada; Redwood City, CA; Los Angeles, CA; Minneapolis, MN; Boca Raton, FL; Dallas, TX. Participant imaging entailed a non-contrast whole-body MRI scan as previously detailed^39^. The brain sequences included sagittal 3D T1 MPRAGE, axial 2D FLAIR, and time-of-flight MRA also detailed in prior work^40,41^. The participants (*n* = 10,134) had an age range of 18–97 years. The individuals scanned were generally healthy, though no formal cognitive evaluations were done.

### Evaluation of smoking

Prior to imaging, participants completed intake questionnaires including (i) Demographic information (age, sex, race), (ii) Medical History (hypertension, type 2 diabetes mellitus), and (iii) self-reported smoking. Participants were requested to report the information required to compute pack years of smoking, specifically the number of packs smoked per day and the number of years smoked. Pack years were then computed by multiplying these two numbers together per standard definition^42^. In this study, smokers were thus defined as participants with any self-reported smoking history (i.e., a non-zero pack-year value), and non-smokers were those who reported zero pack years (never smokers). Based on these responses, the participants were categorized into two groups: the Smoking group (*n* = 3292) and the Non-Smoking group (*n* = 6842).

### MRI volumetry of brain regions

The FastSurfer network was used to quantify brain volumes from 3D T1 scans^43^. FastSurfer is a deep-learning pipeline that has been extensively validated and offers rapid automated analysis of structural MRIs of the human brain. It produces outputs that are compatible with FreeSurfer, facilitating scalable analysis of large datasets and enabling time-sensitive clinical applications such as real-time structure localization during image acquisition or the extraction of quantitative metrics. The FastSurfer Convolutional Neural Network (CNN) consists of three fully connected CNNs, which operate on stacks of coronal, axial, and sagittal 2D slices. Additionally, a final view aggregation is performed, combining the strengths of 3D patches and 2D slices. The FastSurfer CNN segmentation on MRI was trained over 134 participants, ranging in age from 27 to 66, and was utilized to segment 96 distinct regional brain volumes. Prior to inputting the MRI brain volumes into the deep-learning networks, all scans were conformed to standard slice orientation and resolution of 1 mm isotropic.

### MRI volumetric measurement of intracranial volume (ICV)

To account for variations in head size among the subjects, a deep-learning model was trained to segment the ICV. To estimate the total ICV, a set of 60 participants was used based on a similar sample size detailed in prior work^44^, and the intracranial compartment of these individuals was manually annotated. These annotated data were then utilized to train the nnU-Net^45^ for generating the intracranial mask. nnU-Net is a self-adapting method for deep-learning-based medical image segmentation. It encompasses preprocessing, network architecture, training, and post-processing steps. The MITK tool^46^ was used to inspect ICV segmentation quality.

### Statistical analyses

All statistical analyses were conducted using the sklearn and SciPy libraries in Python^47^. Two-sample t-tests were done to compare ICV-adjusted brain volumes between groups categorized as smoking versus non-smoking. Our approach sought to understand the influence of smoking on T1 measured brain structure from three perspectives: (i) Overall tissue classes (gray matter and white matter for example), (ii) Lobar structures as these have broad translational relevance to clinical practice, and (iii) Alzheimer risk regions as this disorder is of particular public health importance and smoking is a recognized risk factor in the 2024 Lancet Commission on Dementia Prevention^3^. The brain regions evaluated from MR neuroimaging thus focused on: Macrostructural Parenchymal Tissue Classes: (i) Total gray matter volume, (ii) Total white matter volume, Lobar volumes: (i) Frontal, (ii) Temporal, (iii) Parietal, (iv) Occipital, and early Alzheimer’s regions: (i) Hippocampus, (ii) Posterior Cingulate gyrus, (iii) Precuneus. Regression analyses were performed in smokers between Pack Years of Smoking and these brain regions in two different models. Model 1 adjusted for age and sex, and study site Model 2 adjusted for age, sex, site, and BMI to determine if potential mediation effects existed from BMI on the results observed in Model 1. We did not adjust for hypertension or diabetes as these variables did not comprise more than 10% of the entire sample. Also, we have previously shown that these variables do not drive brain volume loss in a statistically significant way when compared to BMI^17,48^. A Benjamini–Hochberg False Discovery Rate of 5% was applied to control for multiple comparisons^49^.

## Data Availability

Access to the data analyzed in this article will be considered on a case-by-case request and may be provided upon approval by study leadership.
